# Optimizing the cascade of prevention to protect people from tuberculosis: A potential game changer for reducing global tuberculosis incidence

**DOI:** 10.1371/journal.pgph.0003306

**Published:** 2024-07-02

**Authors:** Alberto Matteelli, Gavin Churchyard, Daniela Cirillo, Saskia den Boon, Dennis Falzon, Yohhei Hamada, Rein M. G. J. Houben, Avinash Kanchar, Afrânio Kritski, Blessina Kumar, Cecily Miller, Dick Menzies, Tiziana Masini

**Affiliations:** 1 Institute of Infectious and Tropical Diseases, WHO Collaborating Centre for Tuberculosis Prevention, University of Brescia, Brescia, Italy; 2 The Aurum Institute, Parktown, South Africa, School of Public Health, University of Witwatersrand, Johannesburg, South Africa; 3 IRCCS San Raffaele Scientific Institute, Milan, Italy; 4 Global Tuberculosis Programme, World Health Organization, Geneva, Switzerland; 5 Research Institute of Tuberculosis, Japan Anti-Tuberculosis Association, Tokyo, Japan; 6 University College London, London, United Kingdom; 7 TB Modelling Group, TB Centre, London School of Hygiene & Tropical Medicine, London, United Kingdom; 8 Rede Brasileira de Pesquisa em Tuberculose, REDE TB, Rio de Janeiro, Brasil; 9 Programa Acadêmico de Tuberculose, Faculdade de Medicina, Universidade Federal do Rio de Janeiro, Rio de Janeiro, Brasil; 10 The Global Coalition of TB Advocates, India; 11 McGill International TB Centre, McGill University, Montreal, Quebec, Canada; 12 Independent consultant, Lucca, Italy; Universidad Peruana Cayetano Heredia, PERU

## Abstract

The provision of tuberculosis preventive treatment is one of the critical interventions to reduce tuberculosis incidence and ultimately eliminate the disease, yet we still miss appropriate tools for an impactful intervention and treatment coverage remains low. We used recent data, epidemiological estimates, and research findings to analyze the challenges of each step of the cascade of tuberculosis prevention that currently delay the strategy implementation. We addressed research gaps and implementation bottlenecks that withhold key actions in tuberculosis case finding, testing for tuberculosis infection, provision of preventive treatment with safer, shorter regimens and supporting people to complete their treatment. Empowering communities to generate demand for preventive therapy and other prevention services in a holistic manner and providing adequate financial support to sustain implementation are essential requirements. The adoption of an effective, universal monitoring and evaluation system is a prerequisite to provide general and granular insight, and to steer progress of the tuberculosis infection strategy at global and local level.

## Introduction

About one fourth of the world’s population is estimated to have been infected with *Mycobacterium tuberculosis* (Mtb) [1, 2]. Since 2015, the World Health Organization (WHO) recommends treating tuberculosis infection (TBI) in populations at higher risk of progression to the disease, within a larger framework of preventive actions envisaged under Pillars 1 and 2 of the WHO End TB Strategy [3]. Modelling suggested that a combination of screening, tuberculosis preventive treatment (TPT) and treatment of TB disease could bring down levels of TB incidence to those envisaged by the End TB Strategy by 2035 [4].

WHO promotes an integrated approach towards the expansion of TPT, by addressing and strengthening each element in the so called “cascade” of prevention, from the identification of target populations to completion of treatment [5].

At the second United Nations High-Level Meeting (UNHLM) on TB held in 2023, Member States committed to provide 45 million people with TPT between 2023 and 2027, with specific targets for people living with HIV and household contacts of all ages [6]. These are the two populations for whom WHO strongly recommends TPT, alongside other people at higher risk of progressing to TB disease [7]. On one hand, the target seems ambitious considering that the TPT targets of the first UNHLM in 2018 were not reached. On the other hand, these targets should be considered a minimum: while they represent a huge improvement upon current service provision, more will be needed to maximize protection that TPT could provide to contacts, people with HIV and other high-risk populations [8]. This signals an urgent need to mobilize human and financial resources to expand TPT services, including through health system strengthening.

In this paper, we describe the challenges and opportunities for optimizing the cascade of TB prevention towards the targets set for 2027 by the UNHLM on TB, and the WHO End TB Strategy. This entails recognizing the population to target in a public health approach, identifying those who are infected, selecting the best treatment options, engaging affected communities, fighting stigma, and establishing a system to monitor and evaluate the program at all steps of the cascade. We based our discussion on current knowledge and latest hypotheses on the epidemiological burden of TBI.

Our manuscript does not intend to cover all components of a TBI control programme, but rather focus on specific key aspects where critical views by experts are shared, with the intent to prompt reflections on potential solutions to foster access to TPT. Issues such as human resource development, although important, are not discussed in this manuscript.

### Understanding the burden of TB infection

Estimates of the global burden of TBI have usually relied on determining the proportion of the population which immune-reacts to Mtb antigens, assuming that TBI is lifelong [2]. Based on these estimates, approximately one in four individuals has been infected with TB, corresponding to 1.8 billion individuals worldwide, with about 1% of the global population estimated to have been infected in the previous two years [2, 9].

Unfortunately, the persistence of viable mycobacteria cannot be measured by any of the current tests in use for TBI (interferon-gamma release assays (IGRAs), tuberculin skin test (TST), antigen-based TST). In addition, these tests cannot distinguish remote from recent infection, nor can they predict who will progress to TB disease. The current TPT strategy focuses on individuals with a high risk of progression to TB disease, including child contacts (TB risk following infection estimated at 16–19% for children aged 0–5 years) [10, 11] and adult contacts (TB risk following infection estimated at 5%) [12]. Therefore, in this situation, a large number of people provided with TPT are exposed to unnecessary drug toxicity, and programs are challenged by unnecessarily high costs. The risk of overtreatment also raises ethical issues and tensions that should not be overlooked [13]. It is challenging to expand TBI treatment coverage without better predictive tests.

Over the past decade, the binary conceptualization of TB as “latent infection” versus “active disease” has been replaced by a spectrum from early infection through subclinical and clinical disease. Moreover, the assumption of lifelong duration of TBI has been questioned and it has been hypothesized that the proportion of TBI cases at risk of reactivation more than 5 years post infection has been overestimated [14–16]. Recent reviews suggest that individuals may self-clear the infection in absence of chemotherapy, thus no longer being at risk of developing TB in absence of re-infection [9, 17]. In a narrative review of epidemiological data from cohorts of individuals who underwent severe immunosuppression, Behr *et al*. suggested that over 90% of individuals who at one point had TBI, do not have viable mycobacteria later in life [9]. Also, recent evaluation of the risk of infection suggests that the number of individuals with recent exposure to Mtb who develop TBI may be up to 10 times higher than assumed [17].

The discovery of a diagnostics test that would distinguish persistent from cleared TBI, or the refinement of tests for progression that are currently under development would play a game-changer role. Currently, the list of target populations for preventive therapy are defined based on the epidemiological risk of progression once infected. Beyond household contacts, these consist of people with immune compromising conditions (i.e. people living with HIV or receiving tumor necrosis factor alpha inhibitors or transplant anti-rejection).

On the other hand, based on observations that, in high-burden settings, substantial transmission occurs outside of the household [18, 19], a TPT strategy with significant population impact would need to expand beyond TPT for household contacts of bacteriologically confirmed TB patients, and include provision of TPT to additional risk groups.

Providing treatment for TBI or early disease to large parts of the population has been shown to rapidly reduce TB burden in North American indigenous populations in the 1960s [20]. Similar efforts examining the potential impact are underway in Viet Nam and Micronesia [21]. These data suggest that a short and sharp intervention of combined screening for the disease and TPT could reduce the annual risk of infection to such low levels to have a lasting effect [20]. However, with current diagnostic and treatment tools, mass preventive treatment is not conceivable.

### The cascade of TB prevention

**Fig 1:**
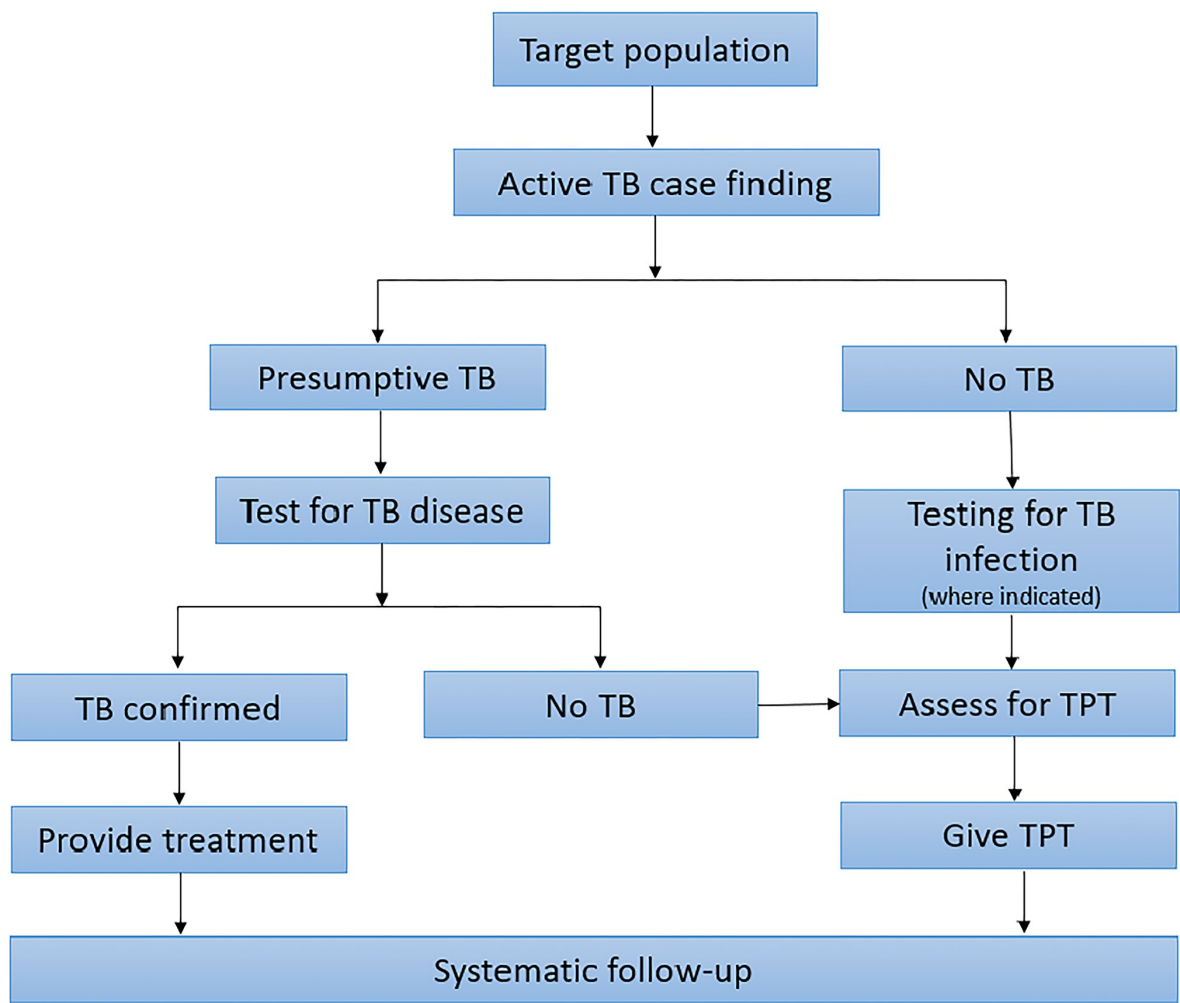
Cascade of TB case finding and preventive treatment.

The “cascade” of TB prevention is a holistic approach to ensure that all individuals at risk of developing TB are systematically identified and provided, if required, access to a full course of TPT. It has four sequential steps: identifying people at risk, excluding TB disease, testing for TB infection and providing TPT and supporting adherence (**Fig 1**) [5]. However, failures at each step may have a cumulative effect, resulting in a small fraction of people in need for TPT benefitting from it.

Systematic reviews that analyzed the TPT cascade indicated that the greatest losses (~50 to 70%) occur in the first steps, during evaluation of people at risk for TB disease and infection [22–24]. Overall, among those estimated to be eligible for TPT, less than 20% complete the entire cascade of care [23]. However, the cascade performance varies by at-risk population and by setting. For example, HIV-positive individuals have higher rates of identification, as well as TPT completion [25], while other studies suggest that only a small proportion of migrants completed TPT [23].

Most studies address the losses in the cascade of prevention in countries with low TB burden. A systematic review and meta-analysis examining interventions to reduce losses in the TBI cascade in 32 countries (only 8 from high TB burden countries), described that patient incentives to complete the initial assessment and to complete the medical evaluation had the largest impact on cascade completion [26]. Capacity building of healthcare workers and implementation of digital solutions led to improved levels of initial assessment, although the latter are still being sporadically used, at least in the WHO European region (Matteelli et al, personal communication). Digital technologies for radiology, though very promising, still lack adequate standardization for immediate roll out. Similarly, home visits and telephone reminders improved the completion of the initial assessment, though to a lesser degree.

Some studies have investigated the role of health systems in the process, the performance of the cascade among adult contacts (over 18 years old) [22, 27], the perception and knowledge of contacts, people with TB and health professionals towards TB transmission and prevention [28, 29], cost-effectiveness and budgetary consequences at the national level, and the role of the private health sector [30].

Even if most studies were carried out in low TB burden settings and results cannot be necessarily translated to high TB burden settings, we can expect losses under programmatic conditions in resource-constrained settings to be even higher. Challenges reported in high TB burden countries include a weak health system infrastructure, knowledge gaps, attitudes and perceptions among patients and providers, stigma, poor access to care, and competing priorities [22–24]. The latter are determined by the absence of symptoms in infected persons who need to take long and potentially toxic medications. Specific challenges to TPT implementation exist for people living with HIV: strong and effective collaboration between TB and HIV programmes needs to be established and maintained [31].

Studies carried out in six high TB burden countries (among which 5 low- and middle-income countries (LMICs) confirmed that a strategy of standardized evaluation, local decision making for improvements, followed by intensive initial and in-service training and implementation of health systems strengthening activities are effective for scaling up TPT [32, 33].

### Target populations

Progress has been made in the past five years for the provision of TPT to people with HIV. Between 2005 and the end of 2022, a cumulative total of 17 million people living with HIV were initiated on TPT [8], equivalent to just under half of the 39 million people estimated to be living with HIV in 2022. However, global TPT coverage remains unsatisfactory. Among 61 countries that reported data, a median of 34% (IQR: 8.8–63%) of people living with HIV who were newly started on antiretroviral therapy (ART) received TPT in 2021 [8]. [UNAIDS epidemiological estimates, 2023 (https://aidsinfo.unaids.org/] At the UN high-level meeting on HIV and AIDS held in June 2021, countries committed to ensuring that 90% of people living with HIV receive TPT by 2025 [34]; however, by 2022, only 52% of them were treated.

TPT coverage remains much lower in contacts of all ages. The cumulative number of contacts initiated on TPT between 2018 and 2022, at 4.2 million, is only 17.5% of the first UNHLM target of 24 million by 2022; this number included 2.2 million children aged under 5 years (55% of the 5-year sub-target of 4 million) and 2.0 million people in older age groups (10% of the 5-year sub-target of 20 million) [8]. The new UNHLM target of reaching 45 million people with TPT by 2027 of whom 30 million household contacts, is therefore ambitious and will require a substantial intensification and expansion of efforts and investment. A recent study estimated that US$ 6.7 billion would have been needed to reach the 2018 UNHLM TB detection and TPT targets in 33 high-TB burden countries, of which US$ 288 million for the systematic screening of household contacts of people with pulmonary TB [35]. Improving TPT provision among household contacts requires more contact evaluation and provision of testing and TPT at household and community level, where community empowerment and engagement is key (**Box 1**) [36].

**Box 1. Communities as key players in addressing TB infection across the cascade of prevention–the example of India**

India’s National Strategic Plan aims to eliminate TB by 2025 [37]. However, TB screening and TPT were, since recently, infrequently performed and administered [38]. In 2021, the National TB Elimination Programme (NTEP) took a significant step towards this ambitious goal by expanding its TPT policy to include all household contacts (HHC) of index pulmonary TB patients irrespective of their age, and other risk groups [39]. The Global Fund has been supporting NTEP by funding substantial projects focused on TPT interventions. Despite these efforts, the TB Annual Report 2022 indicated that only 6% of eligible HHC aged above five years received TPT from July to December 2021.Major challenges for TPT implementation in India include stigma associated with TB and HIV, as well as lack of treatment literacy and community-led mobilization, which are affecting the demand for TPT.

There are several reasons for that: association with TB, and indirectly HIV, commonly attracts stigma. Lack of treatment literacy and community led mobilization is affecting the demand for short course TPT. nadequate information and guidance on the importance of TB prevention through TPT provision affects communities’ willingness to take medications while asymptomatic, resulting in most HHC not taking TPT as prescribed.

To address the widening gap between India’s TPT goals and implementation, it is essential to shift the way TPT is regarded by the community to create demand and decrease stigma. To do so, TPT provision should be explained and seen as an insurance, much like fireproofing a house before a fire. A meaningful engagement of affected community members is prevented by the prevailing medicalized approach to TB that alienates the affected community members and prevents their empowerment. Community based strategies, education and counselling about TPT have all been proved to play an essential role in medication adherence [40]. The gap in TPT access can only be bridged by the community members taking the reins through long term interventions that keep the community at the centre.

Mobilizing and partnering with communities to improve the reach, awareness, acceptability, and sustainability of PMTPT interventions is an essential strategy to generate demand for TPT and other prevention services.

An informed community with treatment literacy is better equipped to generate the demand and overcome stigma. Community-based models of TB screening and TPT management have proven to be feasible and acceptable to beneficiaries and healthcare providers, with a positive impact on the number of contacts screened for TB and started on TPT [41, 42]. A strong communication drive, by the community for the community to promote TPT and allay undue concerns about its safety, effectiveness, and risk of generating drug resistance should complement other efforts.

In general, moving away from facility-based approaches to household-based/family-centred approaches with community-based treatment support can facilitate the implementation of TPT, while also offering the opportunity to promote integration of TB and HIV screening at the community level. However, there is no one-size-fits-all: more research is needed to establish the optimal model for community engagement in different countries and healthcare settings.

Empowering and providing financial support to community volunteers is essential to ensure the successful implementation of all community-based interventions. Therefore, an essential activity of communities will also involve lobbying and advocating with local and national health ministries for allocation of adequate resources for such interventions.

Other people at risk include people who are initiating anti-tumor necrosis factor treatment, receive dialysis, prepare for an organ or hematological transplant, or who have silicosis. For these groups, systematic testing for TB infection and provision of TPT for those who test positive, is also strongly recommended [7]. In addition, systematic TB infection testing, and treatment is conditionally recommended for prisoners, health workers, immigrants from countries with a higher TB burden than the host country, homeless people and people who use drugs [7]. However, for these groups, no global data on coverage is available.

**Fig 2:**
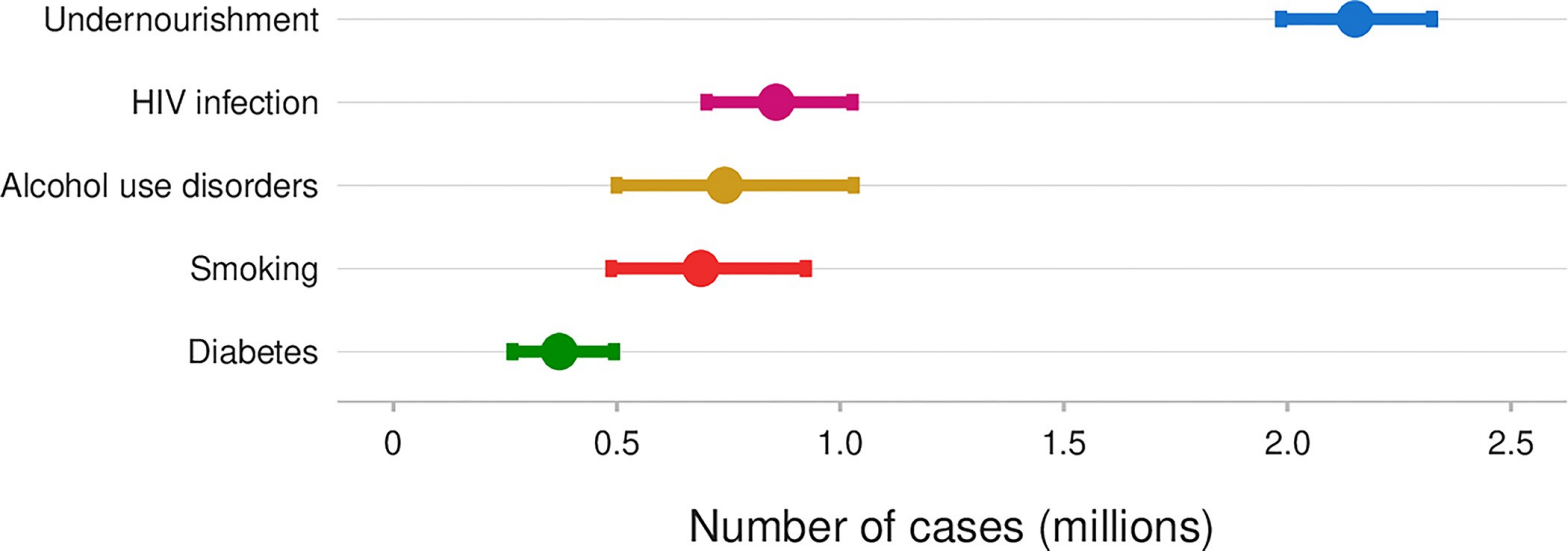
Global estimates of the number of TB cases attributable to selected risk factors, 2022. Sources of data used to produce estimates were: Imtiaz S et al. Eur Resp Jour (2017); Hayashi S et al. Trop Med Int Health (2018); Lönnroth K et al. Lancet (2010); World Bank Sustainable Development Goals Database (http://datatopics.worldbank.org/sdgs/); WHO Global Health Observatory (https://www.who.int/data/gho); and WHO Global TB Report 2023.

Safer and shorter treatments than the ones that we have today combined with new diagnostics that accurately predict risk of disease progression could shift the balance between benefit and harm in favor of treating TB infection in populations at higher risk of TB but for whom systematic testing for TB infection and TPT is currently not recommended (**Table 1**). Indeed, it is estimated that globally in 2022, 2.2 million incident cases of TB were attributed to undernourishment, 0.73 million to alcohol use disorders, 0.70 million to smoking and 0.37 million to diabetes mellitus (**Fig 2**) [8]. However, despite evidence for increased prevalence of TB infection and TB disease in these population groups, there is a paucity of data from clinical trials on the benefits and harm of systematic testing for TB infection and TPT. For people with diabetes, the PROTID trial will provide information on the risk-benefit ratio of TPT, with results expected by 2026 [43]. Certainly, TBI testing and treatment should not be siloed, but rather integrated in the management of diabetes, alcohol use disorders, and in the presence of other risk factors.

**Table 1:** Risk of TB for potential target populations.

Population with risk factors	Risk of TB	Health outcomes related to risk factor	References
Malnourishment	Lower body mass index (BMI) is associated with an increased risk of TB, with a reduction in TB incidence of 13.8% (95% confidence interval: 13.4–14.2) per unit increase in BMI within the range 18.5–30 kg/m2.	There are multiple pathways by which undernourishment can increase the risk of TB, including cell-mediated immunity and micronutrient deficiency.	[44, 45]
Alcohol use disorder	RR from 1.35 to 1.9 associated with alcohol use and a RR from 3 to 3.33 associated with alcohol use disorder. An exposure-response analysis showed that for every 10–20 g of daily alcohol intake, there was a 12% increase in risk.	There is also an increased risk of treatment failure and development of drug-resistant TB.	[46–48]
Smoking	RR ranging from 1.5 to 2.0	Smokers are also at an increased risk of drug-resistant TB and poor outcomes of TB, including relapse and death.	[49–52]
Diabetes mellitus (DM)	RR ranges from 1.5 to 2.0 to 3.1, with a decreasing risk in patients with well-controlled DM.	Patients with DM were also at an increased risk of relapse, treatment failure and death	[53–56]
Mental illnesses (depression and schizophrenia)	Effect estimates ranging from HR = 1.15 (95% CI 1.03 to 1.28) to 2.63 (95% CI 1.74 to 3.96) for depression and HR = 1.52 (95% CI 1.29 to 1.79) to RR = 3.04 for schizophrenia.	Individuals with mental illnesses including depression and schizophrenia experience increased TB incidence	[57]
Previous TB	RR for recurrent TB of 2.0 after first TB episode, and up to 17 after a fifth TB episode.	Patients with a history of TB are at increased risk of subsequent TB episodes, poor outcomes and developing drug-resistant TB. People with CXR changes suggestive of past TB are also at risk of developing a TB episode [58, 59].	[52, 60–64]
Chronic lung disease	Hazard ratios ranging from 2.5 in China to 3.0 in Sweden.	People with chronic obstructive pulmonary disease have an increased risk of developing active TB.	[65, 66]
Substance use disorder	Not available	People with substance use disorder are at increased risks of treatment failure, development of drug resistance, and mortality from TB due to low adherence and coincident clinical socioeconomical and structural risk factors.	[67]
Pregnancy	IRRs for TB in pregnant women of 1.4 and of 1.9 for postpartum women compared with non-pregnant women.	TB in pregnancy is associated with adverse outcomes and complications during birth. These outcomes include a roughly 2-fold increased risk of premature birth, low birthweight and intrauterine growth retardation, and a 6-fold increased risk of perinatal death.	[68–70]

IRR: incidence risk ratio; RR: relative risk

### Tests for TB infection

All the WHO-recommended diagnostics in use for detecting TB infection (TSTs, IGRAs, new skin tests based on RD1 antigens) can identify people that have been exposed to the Mtb complex and have mounted an immunological response to the infection. Unlike TST, the tests based on RD1 antigens (IGRAs and new skin tests) have the advantage of not cross-reacting with bacille Calmette-Guérin (BCG) vaccine and most nontuberculous mycobacteria [71]. Enzyme-linked immunosorbent spot- (ELISpot-) based tests show an increased sensitivity in the immunosuppressed population, but they are complex and have currently limited use in high TB burden countries.

Although IGRAs are unable to predict who among the exposed population will develop the disease, a recent metanalysis by Ledesma et al. [72] underlines that high INF gamma values in response to the “in vitro” stimulation are associated to an increased risk of progression to active TB. This finding could be used to guide the use or preventive treatment. Quantitative reporting is routinely used in the majority of high income, low incidence TB countries where the IGRAs are used to test contacts of people with tuberculosis. Therapy is still offered based on stable conversion, more than on absolute value to children, young adults, and immunosuppressed individuals.

Unfortunately, all tests have a low positive predictive value (PPV) for progression to subclinical or clinical TB, which can lead to unnecessary treatment and, among other things, raises ethical issues [12, 13, 73]. Moreover, the number needed to test to prevent one case of TB is high, significantly lowering the cost-effectiveness of the whole strategy. Until improved tests become available, IGRAs- and TST-based screening approaches should target high-risk groups only.

In the contest of TB elimination, in low TB incidence settings, where chances of reinfection are low to very low, IGRAs and TST retain a role in screening target high-risk groups [74] such as people living with HIV, TB contacts, and the increasing pool of people who are immunosuppressed due to co-morbidities, cancer or iatrogenic causes.

Children exposed to close contacts represent an unresolved challenge. Newborns have an “immature” response to IGRAs and TST [75], and children under five years of age are at higher risk of rapid progression if infected, which justifies the current policy to offer them TPT without testing. Uncertainties remain on how to manage children exposed to resistant forms of TB. In this group, a test able to monitor early signs of progression (now called tests for incipient TB) could find potential applications. Signatures able to predict progression from infection to TB disease have been identified, few being able to meet the targets established by the target product profile (TPPs), and all with a short horizon of one year or less. However, there are few data on the performance of any of these in children and adolescents, overall suggesting lower performance compared to adults [76].

Based on findings from technologies under current evaluation, it is likely that at least one test of progression compliant with the WHO target product profile characteristics—minimal sensitivity of 75% and specificity of 75%—would be on the market in the next five to seven years.

Tests that identify those with TB infection who are at highest risk of disease after a defined exposure episode (also called tests for incipient TB) have been developed and initial results have been promising [77]. Unfortunately, even though one candidate test did identify persons at very high risk of disease, a trial of treatment directed by this test did not show significant reduction in rates of TB disease (CORTIS trial) [77] Further work is required, but the concept of RNA ‘signatures’ as a novel approach to diagnosis of TB infection could result in vastly fewer people receiving TPT.

### TB preventive treatment

After several large-scale trials demonstrated efficacy of isoniazid monotherapy in preventing TB in child contacts [78] and many other populations [79], a six-month regimen of isoniazid preventive treatment (IPT) became of common use in the 70s. Even though isoniazid has been the most widely used regimen for TPT, several factors have limited its uptake, in particular poor adherence due to long duration of treatment and side effects [80, 81].

Over the past two decades, several shorter rifamycin-based regimens have been assessed for safety, tolerability and efficacy.

The three-month regimen of daily isoniazid and rifampicin (3HR) demonstrated high uptake and completion rates among child contacts, as well as low rates of mild adverse events [82] The availability of a dispersible fixed-dose combination (FDC) (HR 75/50), which is already used for children with DS-TB, makes this regimen the best option for children. However, in adults, 3HR has similar levels of hepatotoxicity and similar completion rate as IPT.

The combination of isoniazid and rifapentine given once weekly for 12 weeks (3HP) was shown to be substantially less hepatotoxic and lead to significantly higher completion rates compared to IPT, though it was associated with occurrence of a hypersensitivity systemic immune reaction [83]. On the other hand, a recent metanalysis reported a higher proportion of grade 3 and 4 side effects and a higher discontinuation rate for 3HP compared to 6–9 month IPT [84]. Overall, 3HP is attractive because it increases adherence. A 150 mg scored, dispersible tablet formulation of rifapentine has been developed, which will facilitate 3HP dosing in younger children [85]. Meanwhile, a Rifapentine Crush study is being planned to evaluate the effect of manipulation of the adult tablet on bioavailability to aid the implementation of 3HP in children.

A regimen of one-month daily INH and rifapentine (1HP) is conditionally recommended by WHO for people aged above 13 years but requires further evaluations of safety and efficacy in people without HIV. 1HP is approximately 1.6-fold more expensive than 3HP and the HP 300/300 mg FDC cannot be used given different dose ratios.

The four-month regimen of daily rifampicin (4R) has a very good safety profile [84] but has been poorly implemented so far. This may be due to the perceived risk of development of rifampicin resistance as well as challenges with access to rifampicin capsules.

Remarkable price reductions for rifapentine and the development of the HP FDC (300/300 mg) that has drastically reduced pill burden, have been game changers for the implementation and scale up of rifapentine containing regimen (9 tablets daily at 72 USD/patient course *versus* 3 tablets daily at 14.25 USD/patient course, with a further price reduction to 10 USD/patient course announced in October 2023). Increased manufacturing capacity of rifapentine, from 180,000 patient-courses/year in 2018 to four million in 2023 and two generic manufacturers entering the market, has supported scale-up of 3HP. However, the need to check for impurities, notably nitrosamines, before each batch of rifapentine can be shipped and the limited number of laboratories that can perform quality checks, may create a bottleneck for supplying rifapentine-based products.

Research for newer TPT regimens is ongoing. A six-week daily rifapentine regimen is being investigated in the ASTERoiD trial [86] and two months daily rifampin at double or triple standard dose is being tested in the 2R2 trial [87]. TPPs for TPT regimens have been developed to ensure full alignment between developers’ performance and operational targets for new TPT regimens with the needs of users, including people living with HIV [88]. A mouse model of chronic TB infection in which mice receive Bacille Calmette-Guérin (BCG) vaccination and then are infected with virulent *M*. *tuberculosis*, has shown good ability to predict relative efficacy of different TPT regimens [89] and is being used to identify new candidate regimens.

Before 2024, WHO conditionally recommended TPT for contacts of people with MDR-TB with different treatment options (levofloxacin, ethambutol, ethionamide). Results from two randomized controlled trials of levofloxacin-based TPT among household contacts of people with MDR-TB (TB CHAMP in infants and children under 5 years and V-QUIN in people of all ages) were reviewed by WHO in December 2023 leading to a recommendation to use levofloxacin daily for six months to protect contacts following exposure to MDR-TB [90]. Another trial is currently evaluating delamanid-based TPT (PHOENiX trial) [91].

The absence of a single TPT regimen that meets the needs of all people at risk of TB complicates the roll out of a public health approach strategy. The BREAK TB study plans to assess bedaquiline for TB prevention among adults, children and pregnant women with DS-TB and/or RR-TB exposure [92].

Vaccination with BCG was first introduced in 1921. Since then, no better vaccine has been identified or licensed. However, the M72/AS01E vaccine provided 54.0% protection against pulmonary TB disease in adults with a positive IGRA during an average of 2.3 years follow-up, without evident safety concerns [93]. While 54% efficacy is far less than efficacy of many current vaccines, nevertheless the potential population impact of such a vaccine would be important. Beyond M72/AS01E, the pipeline of vaccines for TB includes many more candidates in different stages of development [8].

### Setting up a monitoring and evaluation system

Effective monitoring and evaluation across the entire TB cascade of prevention is vital to identify potential local bottlenecks and overcome them effectively to ensure scale-up of TPT. One major challenge is that infection is not a notifiable condition in most countries [5, 32, 94]. Recording and reporting of people with TB infection is operationally cumbersome; however, this could initially be prioritized by countries aiming for elimination, and notification could be limited to people in risk groups who have been newly identified so that it increases the likelihood of proper follow up of TPT.

WHO recommends specific indicators to assess the various steps in this process. (**Table 2**) [5]. They include at a minimum, coverage of contact investigation, coverage of TPT, and TPT completion.

**Table 2:** Key monitoring indicators for the programmatic management of TPT.

Indicators	Definition	Numerator	Denominator
Contact investigation Coverage	Number of contacts of bacteriologically confirmed pulmonary TB patients evaluated for TB disease and TB infection out of those eligible, expressed as a percentage	Total number of contacts of bacteriologically confirmed pulmonary TB patients who completed evaluation for TB disease and TB infection during the reporting period	Total number of contacts of bacteriologically confirmed pulmonary TB patients during the reporting period
TPT coverage	Number of individuals initiated on TPT out of those eligible, expressed as a percentage.	Total number of individuals eligible for TPT who initiated treatment during the reporting period	Total number of individuals eligible for TPT during the reporting period
TPT completion	Number of individuals completing TPT out of those initiating treatment, expressed as a percentage	Total number of individuals who completed a course of TPT during the reporting period	Total number of individuals who initiated a course of TPT during the reporting period

Source: WHO operational handbook on tuberculosis. Module 1: prevention—tuberculosis preventive treatment

However, the availability of monitoring and evaluation systems for PMTPT is still very limited. In 2021, among 30 high TB/HIV burden countries, only 13 reported data concerning TPT initiation for HIV-positive individuals while 4 of the 30 high TB burden countries did not report data on contacts starting TB preventive treatment in 2021 [8]. Data on TPT completion are even more sparse, with only 5 high TB burden countries reporting completion among people with HIV started on TPT in 2020 and only 16 for contacts [8].

Globally, data on TPT completion among household contacts were available in 83 countries in 2022 [8]. However, up to now, no country adopted a system to monitor all the cascade of prevention indicators.

It is evident that a sustainable monitoring and evaluation system for TPT will need to be embedded in overall national TB data monitoring efforts. Up to now, global TPT monitoring is reliant on paper-based registers in low- and middle-income countries with a high burden of TB. Since case-based electronic recording and reporting systems for TB are being more and more widely adopted, they could also include pivotal steps of the TBI cascade. Mobile devices can be particularly potent tools to streamline onsite data collection. WHO has developed a mobile application to facilitate this process, allowing for real-time visualization [7]. The challenge will be to establish homogeneous systems worldwide, as well as involving health programmes beyond TB, to include diverse target populations.

Data utilization should be strengthened to showcase the value of programmatic data, which would further motivate the collection of quality data. The COVID-19 pandemic demonstrated the power of real-time dashboards, where anyone, including the public and policymakers, could instantly check case numbers, enhancing national response transparency and accountability. A similar tool for TB programmes would be extremely helpful. For instance, the Philippines has published a real-time TB data dashboard, including on TBI indictors, where data are visualized in easily understandable maps and graphs, allowing disaggregation by sub-national levels [95]. This can ease the workload of program officers, facilitate quarterly data reporting as well as encourage community participation in monitoring and evaluation while promoting accountability. In addition, the use of artificial intelligence may augment the power of electronic monitoring and evaluation. AI may be used, for example, to help identify patterns and trends, aiding local programmes to take immediate action based on data insights [96]. However, the realization of this ambitious vision globally will require more investment in resources and infrastructure for electronic monitoring and evaluation.

## Conclusions

TPT remains a critical intervention to end TB in the foreseeable future. However, despite advancements in increasing TPT coverage in the last few years, the pace of current efforts will not make a sizeable dent in reducing the burden of TB disease.

For further improvement, updated burden estimates are much needed and will likely allow to better focus PMTPT interventions (i.e. on persons with recent TB infection). Cheaper diagnostics that are better prognostic of progression could pave the way for strategies that can be applied to broader segments of the general population, in populations with high TB burden or situations with increased risk of transmission. Making TPT safer and easier to access will enhance coverage amidst larger swathes of the global population at increased risk. In the medium-to-long term, developing a pan-TPT regimen that can be administered irrespective of age and other factors like HIV status could facilitate programmes’ efforts to expand TPT, including facilitating healthcare workers and streamlining procurement. A single dose, slow-release formulation that is cheap and better tolerated than current TPT would make it more feasible to implement broad-based population action in a similar manner to mass deworming programmes in endemic countries or even vaccination.

Moving forward, more studies are needed to evaluate the TBI cascade of care in high TB burden countries, with a focus on the first steps of the cascade to understand challenges and develop strategies to maximize the identification and recruitment of people. Case-based electronic recording and reporting across the TBI cascade will help to understand where the greatest losses are and act accordingly.

Synergies with other measures to curb TB, such as effective vaccines, case finding and treatment and action on social determinants of the disease will remain important. Empowering communities to generate demand for TB preventive services in a holistic manner and providing adequate financial support to sustain implementation will need to be prioritized.
